# Gene Therapy in Mouse Models of Deafness and Balance Dysfunction

**DOI:** 10.3389/fnmol.2018.00300

**Published:** 2018-08-29

**Authors:** Lingyan Wang, J. Beth Kempton, John V. Brigande

**Affiliations:** Oregon Hearing Research Center, Department of Otolaryngology, Oregon Health & Science University, Portland, OR, United States

**Keywords:** gene therapy, congenital deafness, fetal gene transfer, transuterine microinjection, window of therapeutic efficacy

## Abstract

Therapeutic strategies to restore hearing and balance in mouse models of inner ear disease aim to rescue sensory function by gene replacement, augmentation, knock down or knock out. Modalities to achieve therapeutic effects have utilized virus-mediated transfer of wild type genes and small interfering ribonucleic acids; systemic and focal administration of antisense oligonucleotides (ASO) and designer small molecules; and lipid-mediated transfer of Cas 9 ribonucleoprotein (RNP) complexes. This work has established that gene or drug administration to the structurally and functionally immature, early neonatal mouse inner ear prior to hearing onset is a prerequisite for the most robust therapeutic responses. These observations may have significant implications for translating mouse inner ear gene therapies to patients. The human fetus hears by gestational week 19, suggesting that a corollary window of therapeutic efficacy closes early in the second trimester of pregnancy. We hypothesize that fetal therapeutics deployed prior to hearing onset may be the most effective approach to preemptively manage genetic mutations that cause deafness and vestibular dysfunction. We assert that gene therapy studies in higher vertebrate model systems with fetal hearing onset and a comparable acoustic range and sensitivity to that of humans are an essential step to safely and effectively translate murine gene therapies to the clinic.

## Molecular Strategies Underlying Inner Ear Gene Therapy

Gene replacement, augmentation, knock down and knock out strategies have been elucidated to ameliorate the effects of deleterious mutations that cause hearing loss and vestibular dysfunction in mouse models of human inner ear disease. Table 1 presents gene therapies that have been tested for genetic forms of hearing loss and vestibular dysfunction and serves as an organizational tool to structure discussion of the rich diversity of approaches deployed.

**Table 1:** Therapeutic strategies to restore hearing and balance in mouse models of deafness and vestibular dysfunction*.

Common gene name (human gene symbol; deafness locus)	Mouse model	Mutant allele	Therapeutic strategy	Gene or drug delivered	Type of injection	Age at delivery	Phenotypic analyses
Vesicular glutamate transporter 3 (SLC17A8; DFNA25; Ruel et al., 2008)	*Vglut3* KO (Seal et al., 2008)	*EGFP/Neo* cassette into exon 2	gene replacement	AAV2/1-*Vglut3*	RWM or cochleostomy	RWM: P1–P3, P10–P12; cochleostomy: P10–P12	RWM: P1–P3^†^: ABR, CAP, acoustic startle (Akil et al., 2012)
Harmonin (USH1C; DFNB18; Ahmed et al., 2002; Ouyang et al., 2002)	*Ush1c* knock-in (Lentz et al., 2010)	French-Acadian c.G216A	gene augmentation	AAV2/Anc80L65.*CMV.harmonin-b1* (Landegger et al., 2017)	RWM	P0–P1; P10–P12	P0–P1^†^: MET, ABR, DPOAE, acoustic startle (Pan et al., 2017)
			gene augmentation by correction of pre-mRNA splicing	ASO-29	IP	P3–P16	P5^†^: ABR; P3–13^†^: open field, rotations/sec (Lentz et al., 2013)
					IP	Single dose: P1, P5, or P7; multiple doses: P1, 3; P1, 3, 5, 7	P5^†^: ABR and DPOAE (Ponnath et al., 2017)
					IP	P1; P3–P5; P4/5; P1, 3, 5, 7; P15	P1^†^: VsEPs (Vijayakumar et al., 2017)
					TMI into amniotic cavity	E13-E13.5	pre-mRNA splicing correction (Depreux et al., 2016)
Transmembrane channel-like 1 (TMC1; DFNB7/11 for Tmc1^Δ^ and DFNA36 for Tmc1^Bth/+^; Kurima et al., 2002)	*Tmc1*^Δ^ (Kawashima et al., 2011)	*LacZ/Neo* cassette deleting exons 8 and 9.	gene replacement	AAV2/1-*Tmc1*; AAV2/1-*Tmc2*	RWM (bulla intact)	P0–P2	MET, ABR, DPOAE (Askew et al., 2015)
	*Tmc1*^Bth/+^ (Vreugde et al., 2002)	*Beethoven*; missense mutation: p.M412K, c.T1235A	knockout of autosomal dominant disease allele	*S. pyogenes* Cas9–sgRNA ribonucleo–Protein complexes	Cochleostomy or posterior canalostomy	P0–P2: cochleostomy (Tmc1^Bth/+^); 6 wk: canalostomy (*Atoh1-GFP*)	MET, ABR, DPOAE, acoustic startle (Gao et al., 2018)
			knockdown of autosomal dominant disease allele	AAV2/1 and AAV2/9 vectors encoding artificial *siRNA* and *eGFP*	RWM	P0–P2	MET, ABR, DPOAE (Shibata et al., 2016)
Lipoma HMGIC fusion partner-like 5/tetraspan membrane protein of hair cell stereocilia (LHFPL5; DFNB66/67; Tlili et al., 2005; Kalay et al., 2006; Shabbir et al., 2006)	*Lhfpl5* KO (Longo-Guess et al., 2007)	*LacZ/Neo* cassette deleting exons 1 and 2 (van Wijk et al., 2006)	gene replacement	Exosome-AAV2/1-*Lhfpl5*	RWM (György et al., 2017)	P1–P2	ABR, head tossing, circling (György et al., 2017)
Scaffold protein containing Ankyrin repeats and SAM domain (SANS; USH1G; Mustapha et al., 2002; Weil et al., 2003)	*Ush1G* KO (Caberlotto et al., 2011)	*Sans* null: Ush1G^fl/fl^ crossed to PGK-Cre	gene replacement	AAV2/8-*Sans-IRES-GFP*	RWM	P2.5	ABR, circling, VOR (Emptoz et al., 2017)
Whirlin (WHRN; DFNB31; Ebermann et al., 2007)	*Whirler* KO (Whrn^wi/wi^; Lane, 1963; Holme et al., 2002)	592 base pair deletion between exons 6–9	gene replacement	AAV2/8-*whirlin*	Posterior canalostomy	P0–P5	ABR, VsEP, open field, RotaRod, swim (Isgrig et al., 2017)
*Clarin 1* (CLRN1; USH3; Sankila et al., 1995; Joensuu et al., 2001)	KO-TgAC1 (Geng et al., 2012)	*Clarin 1* KO with* Clrn1* expression from *Atoh1 3’enhancer/β-globin* promoter	gene augmentation	AAV2-*smCBA*-*Clrn1* and AAV8*-smCBA-Clrn1* with or without *Clrn 1* UTRs	RWM	P1–P3	click stimulus ABR (Geng et al., 2017)
	Tg;KIKI (Alagramam et al., 2016)	Knock-in of human p.N48K into *Clrn1* locus and *Clrn1* expression from *Atoh1 3’enhancer/β-globin* promoter	Small molecule stabilizer of CLRN1^N48K^	BioFocus 844	IP	P10–P45 (dose escalation paradigm); P30–P45	P10–P45^†^ (ABR; Alagramam et al., 2016)
	*Clrn1* KO (*Clrn1^ex4−/−^)* and conditional KO (*Clrn ^ex4fl/fl^ Myo15-Cre^+/−^*) (Dulon et al., 2018)	*Clrn1* KO: *Clrn ^ex4fl/fl^* crossed to PGK-Cre; Conditional KO: *Clrn ^ex4fl/fl^* crossed to *Myo15-Cre^+/−^*	gene replacement	AAV2/8-*Clrn1*-IRES-GFP	RWM	P1–P3	ABR (Dulon et al., 2018)
Methionine sulfoxide reductase B3 (MSRB3; DFNB74; Waryah et al., 2009; Ahmed et al., 2011)	*MsrB3* KO (Kwon et al., 2014)	*MsrB3* exon 7 replaced with *Neo cassette*	gene replacement	AAV2/1-*MsrB3-GFP*	TMI into otic vesicle	E12.5	ABR (Kim et al., 2016)
Connexin 30 (GJB6; DFNB1B (Del Castillo et al., 2003)	Cx-30 KO (Teubner et al., 2003)	Cx-30 gene replaced with *NLS-β-galactosidase*	gene replacement	*pCMV-Cx-30-GFP*	TMI into otic vesicle with electroporation	E11.5	ABR (Miwa et al., 2013)
	shRNA-Cx-30 in wild type mice	*Cx-30* knock down by four *shRNAs*	gene augmentation	*pU6-shRNA*s and *pCMV-shRNA-resistant Cx-30*	TMI into otic vesicle with electroporation	E11.5	ABR (Miwa et al., 2013)
Connexin 26 (GJB2; DFNB1) (Guilford et al., 1994; Kelsell et al., 1997)	Conditional Cx26 KO (Wang et al., 2009)	*Cx26* null: *Cx26^fl/fl^* (exon 2) crossed to Foxg1-Cre	gene replacement	AAV2/1-*CB7-Gjb2* and AAV2/1-*CB7-Gjb2-GFP*	Cochleostomy	P0–P1	ABR (Yu et al., 2014)
RE1-silencing transcription factor (REST; DFNA27) (Peters et al., 2008)	Rest^+/∆Ex4^ (Nakano et al., 2018)	Rest^+/flEx4^ crossed to Gfi1^+/Cre^ or Rosa^+/CreERT2^	HDAC inhibitors	SAHA (Vorinostat)	Subcutaneous injection	P7–P15, daily.	ABR (Nakano et al., 2018)

*Abbreviations: AAV, adeno-associated virus; ABR, auditory brainstem response; ASO, antisense oligonucleotide; Atoh1, atonal homolog 1; Bth, Beethoven allele; CAP, compound action potential; cDNA, complementary deoxyribonucleic acid; CMV, cytomegalovirus; Cre, Cre recombinase; DFNA, autosomal dominant nonsyndromic hearing loss; DFNB, autosomal recessive nonsyndromic hearing loss; DPOAE, distortion product otoacoustic emissions; E, embryonic day; fl, flanked by loxP sites (floxed); GFP, enhanced green fluorescent protein; IP, intraperitoneal; IRES, internal ribosomal entry site; KO, knockout; LacZ, β-galactosidase; mRNA, messenger ribonucleic acid; MET, mechanotransduction; Neo, neomycin; NLS, nuclear localization signal; p, plasmid; P, postnatal day; RWM, round window membrane; smCBA, small chick β-actin promoter; Tg, transgene; TMI, transuterine microinjection; PGK, phosphoglycerate kinase; shRNA, short hairpin ribonucleic acid; sgRNA, small guide ribonucleic acid; siRNA, small interfering ribonucleic acid; UTR, untranslated region; VOR, vestibulo-ocular reflex; VsEP, vestibular sensory evoked potential. ^†^the age(s) at delivery that produced maximal therapeutic benefit.

### Gene Replacement

The most common approach to address recessively inherited disease genes that create a loss-of-function inner ear phenotype has been to supplant the null disease allele with a wild type copy. The majority of these gene replacement strategies have relied on adeno-associated virus (AAV)-mediated gene transfer to the sensory epithelium by delivery through the round window membrane (Akil et al., 2012; Askew et al., 2015; Shibata et al., 2016; Emptoz et al., 2017; Geng et al., 2017; György et al., 2017; Pan et al., 2017); cochleostomy (Akil et al., 2012; Yu et al., 2014; Gao et al., 2018); or the posterior semicircular canal (Gao et al., 2018; Isgrig et al., 2017). For example, AAV2/1 (pseudotyped AAV vector comprised of the genome of serotype 2 packaged in the capsid from serotype 1) encoding vesicular glutamate transporter 3 (VGLUT3), a protein responsible for transporting the excitatory neurotransmitter glutamate into synaptic vesicles, almost exclusively transduced inner hair cells of the *Vglut3* knockout mouse after round window membrane inoculation at postnatal day 1–3 (P1–3; Seal et al., 2008; Akil et al., 2012). AAV2/1-*Vglut3* gene replacement stably and persistently improved hearing measured by auditory brainstem responses (ABR), compound action potentials (CAP) and acoustic startle (Akil et al., 2012).

### Gene Augmentation

Gene augmentation refers to the creation of wild type gene expression in a background that may exhibit endogenous wild type gene expression that is insufficient for normal function. A gene augmentation approach has been articulated for a recessively inherited null allele that underlies the pathogenesis of Usher syndrome type 1c (USH1C). The French-Acadian mutation (*c.G216A*) introduces a cryptic splice site in exon 3 of the PDZ (postsynaptic density 95; discs large; zonula occludens-1) domain scaffolding protein, harmonin and generates a frameshift with premature stop codon. The splicing machinery strongly prefers the cryptic splice site though wild type splicing is not precluded. The Ush1c *c.G216A* knock-in mouse accurately models the hearing and vestibular abnormalities seen in USH1C patients (Lentz et al., 2007, 2010). A gene augmentation strategy for *Ush1C* has been defined that uses a targeted antisense oligonucleotide (ASO) to interfere with pre-mRNA splicing from the cryptic splice site and enhance wild type splicing. ASO therapy restored functionally relevant levels of protein expression, improved hair cell survival, rescued ABR thresholds at low and intermediate frequencies, and corrected vestibular dysfunction (Lentz et al., 2013; Ponnath et al., 2017; Vijayakumar et al., 2017).

### Gene Knock Down and Knock Out

Knock down and knock out strategies to perturb a dominant mutation that causes inner ear disease have been validated. The *Beethoven* allele (*c.T1235A*) of the transmembrane channel-like 1 (TMC1) gene is dominantly inherited and causes progressive hearing loss in mice (Vreugde et al., 2002). TMC1 is a candidate for the mechanoelectrical transduction channel in sensory hair cells of the inner ear and is required for sensory hair cell function (Kawashima et al., 2011; Pan et al., 2013; Nakanishi et al., 2014). A gene knock down strategy using AAV-mediated transfer of a synthetic small interfering RNA (siRNA) targeting the mutated *Tmc1* message delayed onset of progressive hearing loss in *Beethoven* mice (Shibata et al., 2016). A gene knock out strategy using Cas9 ribonucleoprotein (RNP) complexes targeting the point mutation in *Beethoven* temporarily improved auditory thresholds by an average of 15 dB sound pressure level (dB SPL) from 8 kHz to 22.6 kHz (Gao et al., 2018).

## The Early Neonatal Window of Therapeutic Efficacy in Mice

**Figure 1:**
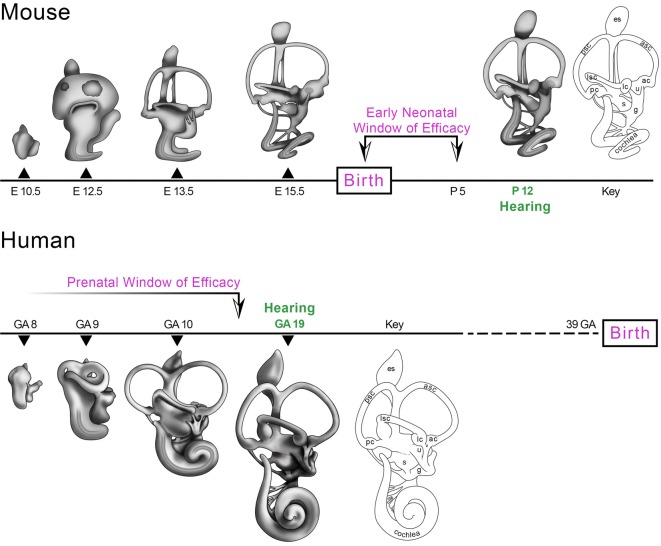
Morphogenesis of the mouse and human inner ears from otic vesicle to the mature membranous labyrinth. The mouse inner ear is structurally and functionally immature at birth with hearing emerging by postnatal day 12 (P12; Shnerson and Willott, 1980). The gene and pharmacotherapeutic strategies identified in Table 1 with the cross symbol compared efficacy at different ages of delivery and suggest that the most effective age to intervene is at P0-P5 prior to the onset of hearing (Early Neonatal Window of Efficacy). The human inner ear is capable of auditory function as early as 19 weeks gestational age (19 GA) when a startle response to low frequency stimuli is present (Hepper and Shahidullah, 1994; Shahidullah and Hepper, 1994). A corollary window of therapeutic efficacy in humans is predicted to close by about GA 18 prior to the onset of hearing (Prenatal Window of Efficacy). There is no human fetal data to set the early boundary of the Prenatal Window of Efficacy and this is represented by the timeline fading to white by GA8. Abbreviations: ac, anterior crista; asc, anterior semicircular canal; g, ganglion; GA, weeks gestational age; E, embryonic day; es, endolymphatic sac; lc, lateral crista; lsc, lateral semicircular canal; P, postnatal day; pc, posterior crista; psc, posterior semicircular canal; s, saccule; u, utricle. Credits: the mouse artwork was modeled after paint fills from Doris K. Wu (Morsli et al., 1998). The human artwork was modeled after George L. Streeter’s drawings (Streeter, 1906).

The ability to microinject bioactive reagents directly into the early neonatal mouse inner ear without significantly affecting the onset or sensitivity of hearing and balance has enabled recent advances in the field of inner ear gene therapy. The plasticity of the early neonatal mouse inner ear to the introduction of aqueous solutions harboring AAV particles, ASOs and Cas9 RNP complexes may be related to its functionally immature state with hearing first emerging only late in the second postnatal week (Shnerson and Willott, 1980). An analysis follows of the ages at which therapeutic interventions have been successfully deployed to rescue hearing and balance in mouse models of genetic hearing loss and vestibular dysfunction. The data define a critical period of intervention after which therapeutic benefits are significantly diminished or lost. We refer to this critical period in mice as the early neonatal window of therapeutic efficacy (Figure 1, Mouse).

### Transmembrane Channel-Like 1

The responsivity of early neonatal hair cells to genetic correction is highlighted by therapies targeting genes required for mechanotransduction. The *Tmc1* and *Tmc2* genes are multi-pass transmembrane proteins whose structure is consistent with channel, receptor, or pore identity (Kawashima et al., 2011; Beurg et al., 2015). *Tmc1* and *Tmc2* are candidates for the mechanotransduction channel in sensory hair cells that open in response to displacement of the stereociliary bundle by the energy in sound or motion (Pan et al., 2013). *Tmc1* knockout mice are deaf and *Tmc1* and *Tmc2* double knockout mice are deaf with vestibular dysfunction (Kawashima et al., 2011, 2015; Nakanishi et al., 2014). Round window membrane injection of AAV2/1-*Tmc1* into the cochleae of *Tmc1* knockout mice at P0–P2 modestly improved ABR thresholds but did not restore distortion product otoacoustic emissions (DPOAE) thresholds, a sensitive measure of outer hair cell function (Askew et al., 2015). The low transduction efficiency of outer hair cells was thought to underlie the inability to fully rescue hearing thresholds. Importantly, the data indicate that AAV-mediated gene transfer to *Tmc1* knockout hair cells at P0–P2 defined a distinct capacity for functional recovery.

### Lipoma HMGIC Fusion Partner-Like 5/Tetraspan Membrane Protein of Hair Cell Stereocilia

The lipoma HMGIC fusion partner-like 5/tetraspan membrane protein of hair cell stereocilia (LHFPL5) protein joins *Tmc1* and *Tmc2* as essential constituents of the mechanotransduction complex (Xiong et al., 2012). *Lhfpl5* knockout mice are deaf with profound vestibular dysfunction (Longo-Guess et al., 2007; Xiong et al., 2012; Beurg et al., 2015). A novel modification of conventional AAV-mediated gene transfer was defined to partially rescue hearing and balance in the *Lhfpl5* mutant mice. Exosomes are membrane bound structures originating from endocytic compartments that contain a diverse array of molecules that mediate intercellular communication (Sarko and McKinney, 2017). The cargo carrying capacity of exosomes has led to their use as vehicles for delivery of therapeutic agents including AAV particles (Fitzpatrick et al., 2014; György et al., 2015; György and Maguire, 2018). Round window membrane injection of AAV2/1 encapsulated in exosomes into the cochleae of *Lhfpl5* knockout mice at P1-P2 modestly improved ABR thresholds and vestibular function assessed by quantifying head tossing and circling behaviors (György et al., 2017). The presence of a potentially interfering hemagglutinin tag on the *Lhfpl5* cDNA and the relatively late delivery of the gene with respect to the onset of hair cell pathology in *Lhfpl5* mutants are leading hypotheses to explain the incomplete hearing recovery observed. However, the extent of vestibular recovery and the modest rescue of ABR thresholds with suboptimal transduction of outer hair cells again suggested that neonatal auditory and vestibular hair cells bear a molecular plasticity that can be biased toward functional repair.

### Scaffold Protein Containing Ankyrin Repeats and Sterile Alpha Motif Domain

Virus-mediated gene replacement approaches for two Usher syndrome genes further demonstrate the plasticity of neonatal hair cells to respond to molecular correction. Scaffold protein containing ankyrin repeats and sterile alpha motif domain (SANS; Kikkawa et al., 2003) interacts with harmonin to form a stable complex that associates with additional bundle proteins to support stereocilia development and function (Yan et al., 2010; Caberlotto et al., 2011; Zou et al., 2017). Mutations in *SANS* cause Usher syndrome type 1G (Ush1g) characterized by congenital profound sensorineural hearing loss and vestibular dysfunction at birth with onset of retinitis pigmentosa by adolescence (Weil et al., 2003). The *Ush1g* knockout mouse is profoundly deaf with vestibular dysfunction characterized by circling and head tossing behaviors (Caberlotto et al., 2011). Round window membrane injection of AAV2/8 encoding *Sans*-*internal ribosomal entry site (IRES)*-*GFP* at P2.5 improved stereociliary bundle morphology in auditory and vestibular hair cells. In addition, *Sans* rescued mechanoelectrical transduction currents in inner and outer hair cells, modestly improved ABR thresholds from 5 kHz to 15 kHz, and completely restored vestibular function measured by circling behavior and vestibulo-ocular reflex (VOR) testing (Emptoz et al., 2017). The data suggest that differential AAV2/8 transduction efficiencies along the tonotopic axis were responsible for the variation in ABR threshold improvement. Similar to AAV2/1, maximal therapeutic benefit with AAV2/8 was achieved by inoculation of the early neonatal mouse cochlea.

### Whirlin

Whirlin is a PDZ domain-containing protein that interacts with myosin 15a, myosin 7a, usherin and adhesion G protein-coupled receptor V1 and is required for stereociliary bundle elongation and stability (van Wijk et al., 2006). Diverse mutations in *WHRN* are associated with DFNB31, an autosomal recessive form of hearing loss, as well as Usher syndrome type 2D (Mburu et al., 2003; Tlili et al., 2005; Ebermann et al., 2007). The *whirler* knockout mouse models DNFB31 nonsyndromic deafness and displays shortened auditory and vestibular stereocilia and is deaf with circling and head bobbing behaviors (Lane, 1963; Holme et al., 2002). Initial work with AAV2/8 encoding whirlin delivered by round window membrane injection at P1–P5 enhanced inner hair cell survival in the cochlear base, improved stereociliary length, and normalized the number of bundle rows, but no improvement in ABR thresholds was achieved (Chien et al., 2016). In subsequent work, AAV2/8 whirlin was introduced by posterior canal inoculation at P0-P5 improving stereociliary bundle length as well as open field, swimming and Rotarod behaviors (Isgrig et al., 2017). Importantly, vestibular sensory evoked potential (VsEP) thresholds were detected in treated mutants with unilateral or bilateral inoculation, with bilaterally inoculated mutants showing improvements in P1-N1 amplitudes and P1 latencies. Furthermore, detectable ABR thresholds were observed in a subset of treated mutants demonstrating a modest improvement of auditory function. The data again suggest that hair cell transduction efficiency is essential for therapeutic benefit with 71%–81% of inner hair cells transduced by posterior semicircular canal administration in those mice with demonstrable ABR thresholds vs. ~15% transduction efficiency by round window membrane inoculation in mice with no hearing recovery (Isgrig et al., 2017). This work is consistent with the previously discussed AAV2/1 and AAV8-based gene therapy studies showing that improvement in sensory function is linked to transduction efficiency of the early neonatal inner ear.

### Vesicular Glutamate Transporter 3

A clearer idea of the age range over which the neonatal cochlea most effectively responds to virus-mediated gene replacement was provided by work in the *Vglut3* knockout mouse. *Vglut3* expression is present in inner hair cells and *Vglut3* knockout mice are deaf due to the absence of glutamate release at synapses with spiral ganglion neurons (Seal et al., 2008). AAV2/1-*Vglut3* was delivered to the cochlea by round window membrane injection at P1–P3 and P10–P12, and by cochleostomy at P10–P12 (Akil et al., 2012). Fortuitously, *Vglut3* was expressed in effectively all inner hair cells along the tonotopic axis of the cochlea regardless of the injection method. Hearing recovery measured by the percentage of animals with ABR thresholds within 10 dB of wild type was more robust through 28 weeks for P10–P12 cochleostomy than for P10–P12 round window membrane inoculation. Importantly, optimal hearing recovery through 28 weeks was achieved by P1–P3 round window membrane injection (Akil et al., 2012). The P1–P3 immature inner hair cells in the *Vglut3* knockout mouse appear to assimilate the effects of gene replacement more readily to achieve maximal auditory function whereas more mature P10 hair cells are less responsive. The data strongly suggest that the early neonatal window of efficacy in the *Vglut3* knockout mouse has started to close before P10.

### Usher Syndrome Type 1c

#### Synthetic AAV: Auditory and Vestibular Rescue

Further corroboration of the optimal timing for early neonatal gene therapy in the *Ush1c* mutant mouse arose from work with an artificial AAV capsid. An AAV2 vector bearing the Anc80L65 synthetic capsid (Zinn et al., 2015; Landegger et al., 2017; Suzuki et al., 2017) encoding *harmonin b1* was injected into the *Ush1c* mutant inner ear through the round window membrane at P0–P1 (Pan et al., 2017). AAV2/Anc80L65-mediated gene therapy restored mechanotransduction in *Ush1c* mutant inner hair cells and vestibular hair cells, rescued ABR thresholds from 5.6 kHz to 22.6 kHz, and improved DPOAE thresholds at low frequencies (Pan et al., 2017). In addition, open field behavior, Rotarod latency to fall, and acoustic startle were all improved. The data suggest that early neonatal gene augmentation by AAV-mediated transduction of sensory hair cells restores mechanoelectrical response profiles and systems-level sensory function. However, administration of AAV2/Anc80L65-*harmonin b1* at P10–P12 in *Ush1c* mutant mice failed to restore ABR and DPOAE thresholds (Pan et al., 2017). Consummate with AAV2/1-*Vglut3* gene replacement outcomes achieved by P0–P1 administration to the *Vglut3* knockout mouse, AAV2/Anc80L65-*harmonin b* inoculation of the Ush1c mutant at P0–P1 was most effective.

#### Antisense Oligonucleotide-29: Auditory Rescue

Pharmacotherapeutic strategies to treat congenital hearing loss and vestibular dysfunction in the *Ush1c* mutant mouse are also limited by a temporally rigid window of early neonatal responsiveness. ASO-29 recognizes the mutant pre-mRNA, sterically blocks the cryptic splice site, and switches splicing toward production of wild type messenger ribonucleic acid (mRNA; Lentz et al., 2013). In initial studies, intraperitoneal injection of ASO-29 at P5 rescued ABR thresholds at low and intermediate frequencies and improved high frequency thresholds modestly (Lentz et al., 2013). Notably, ASO-29 administration at P10 was dramatically less effective with greater than 30 dB SPL elevation in ABR thresholds at all frequencies tested (Lentz et al., 2013). Subsequent work demonstrated that a single dose of ASO-29 at P1 or multiple doses at P1 and P3 improved both ABR and DPOAE responses, while ABR thresholds alone were improved by a single dose delivered at P5 (Ponnath et al., 2017). These data suggest that there are critical epochs during the early neonatal maturation of inner and outer hair cells that are acutely and differentially responsive to ASO-mediated correction of harmonin pre-mRNA splicing.

#### Antisense Oligonucleotide-29: Vestibular Rescue

A stringent therapeutic window of efficacy was also defined for the amelioration of vestibular dysfunction by ASO pharmacotherapy in the *Ush1c* mutant. Intraperitoneal injection of ASO-29 from P3–P13 corrected open field and circling behaviors in mutant mice while dosing at P16 failed to restore wild type behaviors (Lentz et al., 2013). Subsequent work using VsEP to measure vestibular function directly showed that mutant mice had normal response thresholds, latencies, and amplitudes when ASO-29 was delivered at P1 but elevated thresholds with delivery at P5 (Vijayakumar et al., 2017). Significantly, ASO-29 dosing at P15 was not effective at restoring VsEP thresholds. In summary, maximal therapeutic benefit for auditory and vestibular function in the *Ush1c* mutant was achieved by ASO-29 dosing at P1 with diminished or lost benefit by dosing at P5 or later (Ponnath et al., 2017; Vijayakumar et al., 2017).

## Beethoven Allele of Transmembrane Channel-Like 1

AAV2/1-, exosome AAV2/1, AAV8-, AAV2/8-, AAV2/Anc80L65- and ASO-29-mediated gene therapies consistently define P0–P5 hair cells as optimally responsive targets to achieve genetic correction and systems-level functional improvement. A novel intervention using the clustered regularly interspaced short palindromic repeats (CRISPR)/Cas9 nuclease genome editing system further validates the early neonatal window of efficacy while offering potential for adult inner ear genetic modulation. The *Beethoven* missense mutation in the *Tmc1* gene differs in only one base pair from the wild type allele (*c.T1235A*, Vreugde et al., 2002) and the orthologous mutation in humans causes progressive post-lingual sensorineural hearing loss (Kurima et al., 2002; Zhao et al., 2014). The *Beethoven* allele in mice triggers progressive elevation of auditory response thresholds and progressive hair cell loss starting at P30 (Vreugde et al., 2002). A CRISPR/Cas9 strategy was devised to disrupt the *Beethoven* allele of *Tmc1* allowing corrective expression from the wild type allele. Cas9 RNPs harboring a small guide RNA (sgRNA) designed against the *Beethoven* allele was delivered by lipid-mediated transfer to the mouse inner ear at P0–P2 (Gao et al., 2018). Inner hair cell mechanotransduction currents were improved at 2 and 3 weeks post-injection and inner hair cell survival was significantly improved 8 weeks after injection at cochlear positions corresponding to 16–45.25 kHz (Gao et al., 2018). Importantly, ABR thresholds were improved by an average of 15 dB SPL at low through intermediate frequencies 1 month after injection. However, ABR thresholds in treated mice elevated by more than 10 dB SPL at 2 months post-treatment possibly due to confounding effects of uncorrected hair cells. Importantly, a Cas9 RNP targeting GFP was introduced by posterior canalostomy to the *Atonal homolog 1* *(Atoh1)*-*GFP* mouse cochlea at 6 weeks of age to test the efficiency of inner ear genome editing in adult hair cells. Encouragingly, GFP expression was perturbed in 25% of the adult, *Atoh1-GFP* hair cells (Gao et al., 2018). However, validation that CRISPR/Cas9 strategies can be effective in treating a genetic form of deafness or vestibular dysfunction by targeting in the adult inner ear is outstanding. These data affirm the early neonatal window of efficacy observed with AAV- and ASO-based modalities directed against recessively inherited deafness mutations. The data also suggest that improvement in CRISPR/Cas9 targeting efficiency will likely enhance the degree and stability of therapeutic outcomes.

## Adult Inner Ear Responsivity to Therapeutic Intervention

The literature discussed thus far demonstrates that AAV-, ASO- and CRISPR/Cas9 RNP-based therapies achieve maximal benefit for recessively and dominantly inherited forms of deafness and vestibular dysfunction when initiated from P0–P5. Furthermore, initiating therapeutic strategies after P5 shows reduced efficacy or the elimination of therapeutic benefit entirely (Table 1). The thought that the juvenile or adult inner ear may be intrinsically resistant to gene therapy is addressed by the following two studies.

### Clarin 1

Clarin 1 is a four-pass transmembrane protein that is implicated in structural support and compartmentalization of cell membranes by interaction with integrins, ion channels and tetraspanins (Adato et al., 2002; Hemler, 2005; Charrin et al., 2014). Mutations in CLRN1 cause Usher syndrome type 3 characterized by progressive post-lingual hearing impairment and vision loss with variable vestibular involvement (Sankila et al., 1995; Joensuu et al., 2001). *Clarin 1* is expressed in inner and outer hair cells as well as spiral ganglion neurons in the mouse cochlea (Geng et al., 2009). Knockout mouse models of *Clarin 1* show defects in stereociliary bundle patterning and mechanoelectrical transduction with rapidly progressing deafness (Geng et al., 2012). To delay hair cell degeneration and better recapitulate the progressive hearing loss seen in USH3 patients with a missense mutation in p.N48K, *Clarin1* was introduced into the *Clrn1*^N48K/N48K^ (KI/KI) mouse under the control of the *Atoh1* 3’ enhancer/β-globin basal promoter sequence (Chen et al., 2002; Lumpkin et al., 2003; Alagramam et al., 2016). In this *Clarin 1* transgenic model termed Tg;KIKI, *Clarin 1* expression is expected to turn off by about P5 under the *Atoh1/β-globin* promoter. Consistent with this prediction, Tg;KIKI mice showed delayed-onset progressive hearing loss from P22-P70 compared to homozygous knock-in mice that displayed profound hearing loss by P22 (Alagramam et al., 2016).

The p.N48K mutation is hypothesized to reduce surface expression of CLRN1 (Alagramam et al., 2016). BioFocus 844 (BF844), a small molecule emerging from a three step drug development scheme, selectively stabilizes CLRN1^N48K^ and does not operate by general inhibition of proteasomes. Postnatal day 55 Tg; KIKI mice treated with a multi-dose BF844 regimen beginning at P30 showed 27.5–35.0 dB SPL lower ABR thresholds at 8, 16 and 32 kHz compared to untreated mutants. These data suggest that CLRN1^N48K^ stabilization in adult hair cells can restore sensory function to clinically relevant levels. In addition, an escalating multi-dose BF844 regimen beginning at P10 showed an average of 45 dB SPL lower ABR thresholds in the treated mutant across the three frequencies. These data suggest that neonatal hair cells prior to hearing onset are maximally responsive to BF844 pharmacotherapy. In summary, the data critically show that adult hair cells can respond productively to therapeutic intervention though treatment before hearing onset leads to improved outcomes.

### Noise-Induced Hearing Loss in the Guinea Pig

Notch signaling is responsible for lateral inhibition in the developing inner ear which establishes the stereotyped hair cell and supporting cell mosaic essential for sensory function (Lanford et al., 1999; Zine et al., 2000; Petrovic et al., 2014). Notch target genes of the Hairy and Enhancer of split (*Hes*) family are potent transcriptional repressors of *Atoh1* expression preventing hair cell fate commitment (Bermingham et al., 1999; Petrovic et al., 2015). Exposure of the juvenile (200–250 g) Guinea pigs to 125 dB SPL octave-band noise centered at 4 kHz for 3 h results in permanently elevated ABR thresholds and hair cell loss in the basal and first turn of the cochlea (Hirose et al., 2008). Administration of sustained release siRNAs targeting *Hes1* by mini-osmotic pump infusion through a cochleostomy lateral to the round window 3 days after sound exposure significantly improved ABR thresholds on average from 10.5 dB SPL to 13.2 dB SPL at 2–16 kHz (Du et al., 2018). The intervention significantly increased the number of inner and outer hair cells, generated ectopic Myo7a-positive cells morphologically consistent with immature hair cells, and produced both immature and dysmorphogenic bundles assessed by scanning electron microscopy. Importantly, the number of supporting cells decreased along the tonotopic axis, consistent with the interpretation that supporting cells unconstrained by *Hes1*-mediated repression of *Atoh1* contributed to *de novo* hair cell production (Du et al., 2018). These data suggest that a temporary gene knockdown of *Hes1* proximal to a deafening acoustic insult has the potential to modestly restore auditory function in the adult inner ear. It would be important to learn if treatment a month or longer after noise exposure is therapeutic or if a window of efficacy closes soon after the acoustic trauma. The Tg;KI/KI and *Hes1* studies are important counterexamples suggesting that genetic intervention in the diseased or damaged mature inner ear can significantly improve sensory function.

## Implications of the Early Neonatal Window of Efficacy

Gene and pharmacotherapy studies in mice clearly establish the need to deploy corrective strategies in the early neonatal inner ear from P0-P5 for the most effective sensory rescue. The diversity of murine deafness mutants interrogated and the consistency of the data validating the early neonatal window of efficacy provide a firm basis to conceptualize when therapeutic strategies may be most effectively deployed in human patients. To frame this discussion, the morphogenesis of the mouse and human inner ears is briefly reviewed in reference to the onset of hearing in both species.

### Morphogenesis of the Mouse and Human Inner Ears

The inner ear develops from a patch of head ectoderm called the otic placode that invaginates into the mesenchyme forming sequentially a pit, cup and fluid-filled otic vesicle (Figure 1; O’Rahilly, 1963; Sher, 1971; Anniko and Wikström, 1984; Morsli et al., 1998). The otic vesicle and nascent ganglion are present at embryonic day 10 (E10) in mice and by 6 weeks gestational age (GA6; weeks since the first day of the last menstrual period) in humans. The otic vesicle expands in volume with the semicircular canal pouches emerging dorsally and the cochlear duct ventrally. The lateral canal in mice is patent by E12.5 and the anterior and posterior canal plates have begun fusing at this stage which is comparable to GA9 in the human fetus (Streeter, 1906; Sher, 1971). Over the next day in mice and the next week in humans, the fused regions of the anterior and posterior canal plates clear and the cochlear duct nearly completes its first full turn. Auditory hair cells are born starting at E13.5 in the mouse (Cai et al., 2013; Chonko et al., 2013) and inner hair cells are present at GA12 and outer hair cells by GA14 in humans (Locher et al., 2013). The gross morphogenesis of the mouse inner ear is largely complete before birth while the membranous labyrinth of the GA19 fetal human inner ear has reached a comparable stage (Figure 1).

### The Onset of Hearing

The acoustic startle reflex produces contraction of the major muscle groups of the body in response to brief, intense sound and is a behavioral correlate of auditory function. In mice, the musculoskeletal response to intense sound is biphasic with extension of the fore limbs and hind limbs followed by postural flexion (Wilson and Groves, 1972; Davis, 1980). Mice hear as evidenced by startle responses beginning at P12 (Figure 1; Shnerson and Willott, 1980). Human fetal hearing has been assessed by ultrasound-based detection of startle responses to pure tone stimuli delivered by a loudspeaker placed on the maternal abdomen (Hepper and Shahidullah, 1994). A human fetus startled in response to a 500 Hz stimulus at GA19 and all fetuses responded to 500 and 3,000 Hz tones at GA33 (Figure 1; Hepper and Shahidullah, 1994). The startle data establish that mice and humans have a fundamental and profound temporal difference in the onset of hearing. The mouse inner ear is functionally immature at birth with hearing onset only during the second week of postnatal life. Hearing in humans is a distinctly fetal sensory capability with sensitivity to low frequencies emerging early during the second trimester of pregnancy. This fundamental difference in the ontogeny of mouse and human hearing, coupled to the stringency of the early neonatal window of therapeutic efficacy in mice, leads to testable predictions about the timing required to achieve the most complete sensory rescue in human subjects.

## The Prenatal Window of Efficacy in Humans

Mouse gene therapy studies indicate that maximal therapeutic benefit is achieved by intervention at P0-P5 prior to hearing onset at P12. A corollary window of therapeutic efficacy in humans is predicted to close prenatally prior to the onset of hearing at GA19 during the second trimester of pregnancy (Figure 1). The central hypothesis is that the immature prenatal human inner ear, like the immature postnatal mouse inner ear, may be ideally responsive to gene and pharmacotherapeutic interventions. Several studies conducted in the mouse begin to address the feasibility of fetal therapeutics to treat congenital inner ear disease.

### Methionine Sulfoxide Reductase B3

The sulfur atom in methionine is acutely susceptible to oxidation by reactive oxygen species generating methionine sulfoxides (Kim et al., 2014). The reduction of methionine-R-sulfoxide to methionine is carried out by the protein maintenance enzyme methionine sulfoxide reductase B3 (MSRB3; Tarrago et al., 2012; Kim et al., 2014). *MsrB3* is expressed in the mouse auditory sensory epithelium at E15.5 and is enriched beneath the stereociliary bundles of auditory hair cells (Kwon et al., 2014). *MsrB3* knockout in mice results in congenital deafness associated with progressive bundle deterioration and apoptotic cell death. Mutations in *MSRB3* result in prelingual autosomal recessive nonsyndromic hearing loss (Waryah et al., 2009; Ahmed et al., 2011). Microinjection of AAV2/1-*MsrB3-GFP* into the E12.5 otic vesicle established significant but reduced inner ear *MsrB3* mRNA expression compared to wild type levels at P28 (Kim et al., 2016). MsrB3 protein levels were restored to 20% of wild type levels in the inner ears of treated mutant mice and the enzyme immunolocalized to auditory hair cells. Inner and outer hair cell stereociliary bundle morphology was restored to wild type patterns. Remarkably, ABR thresholds were rescued to wild type levels at 4 and 8 kHz with significant but small threshold elevations at 16 and 32 kHz at P30. Deterioration in ABR thresholds after 4 weeks was correlated with bundle degradation. The data clearly establish that AAV-mediated gene transfer to the otic vesicle can temporarily restore hearing in the *MsrB3* mutant. This work suggests that fetal gene transfer is a viable approach to treat congenital hearing loss.

### Gap Junction Protein Beta-6

Gap junctions form intercellular conduits that permit exchange of ions, small metabolites and signaling molecules between the cytoplasm of coupled cells (Beyer and Berthoud, 2018). Six connexins assemble to form a hemichannel in the plasma membrane and hemichannels of adjacent cells align to establish the functional pore (Meşe et al., 2007). Gap junction protein beta-6 (*GJB6*, also known as* CX30*) is one of four connexins expressed in the inner ear that link all supporting cells with adjacent epithelial cells but not hair cells (Nickel and Forge, 2008; Jagger and Forge, 2015). About 1 in 30 individuals with severe to moderate, nonsyndromic hearing loss carries a *GJB6* deletion (Del Castillo et al., 2003). The *Cx-30* knockout mouse fails to establish the endocochlear potential (EP) and the auditory sensory epithelium degrades at P18 (Teubner et al., 2003). A plasmid encoding *Cx-30* fused to *GFP* was microinjected through the uterus into the otic vesicle at E11.5 and subsequently electroporated into the otic epithelium (Miwa et al., 2013). At P30, ABR thresholds at 4, 12 and 20 kHz were restored to control levels (*n* = 4 mutant mice) and the EP was significantly improved (*n* = 5 mutant mice). An alternative model system was developed due to the difficulty in obtaining sufficient numbers of mutant mice for extensive analysis. A plasmid encoding four short hairpin RNAs (shRNAs) against *Cx-30* was electroporated into wild type mice at E11.5 that knocked down *Cx-30*, elevated ABR thresholds to over 90 dB SPL from 4 KHz to 20 KHz, and depleted the EP. Co-electroporation of a shRNA-resistant sequence encoding *Cx-30* along with the shRNAs against wild type *Cx-30* in wild type mice resulted in improved ABR thresholds and EP (Miwa et al., 2013). The data suggest that otic epithelial precursors are amendable targets for electroporation-mediated gene transfer and that transgene bioactivity may sustain a therapeutic response.

### Usher Syndrome Type 1c

While direct injection of bioactive reagents into the developing inner ear is formally a plausible gene therapy strategy, it is unlikely that targeted injections will be conducted into the human otic vesicle which first forms by GA 6 not long after most pregnancies are first verified (Streeter, 1906; O’Rahilly, 1963, 1983). The fluid-filled amniotic cavity immediately surrounds the developing embryo (Pereira et al., 2011) and represents a potential reservoir for therapeutic reagents. Metastasis associated lung adenocarcinoma transcript 1 (*MALAT1*) is a nuclear-localized, long noncoding RNA robustly expressed in a diverse array of tissues that is nonessential for mouse pre- and postnatal development (Zhang et al., 2012). A highly active, systemically-delivered gapmer ASO targeting *MALAT1* RNA effectively reduced expression in most mouse tissues analyzed (Wheeler et al., 2012; Arun et al., 2016). Transuterine microinjection of the *MALAT1* ASO into the E13-E13.5 mouse amniotic cavity reduced *MALAT1* RNA abundance in the inner ear through P15 establishing that the ASO gains entry into embryonic tissues and exerts its bioactive effects through the second postnatal week (Depreux et al., 2016). Furthermore, transuterine microinjection of ASO-29 into the amniotic cavity of E13-E13.5 *Ush1c* mutant mice generated correctly spliced harmonin RNA in the inner ear at P22 (Depreux et al., 2016). These data suggest that amniotic cavity administration of pharmacotherapeutic drugs may be a viable approach to manipulating gene expression in the inner ear through early postnatal stages, though pharmacologic correction of an inner ear phenotype remains to be demonstrated.

## Conclusion

The therapeutic modulation of inner ear gene expression by systemic and focal injections of bioactive reagents has defined authentic gene therapy strategies in mouse models of hearing loss and vestibular dysfunction. The literature establishes that therapeutic intervention in the functionally immature mouse inner ear by 5 days postnatal age leads to optimal recovery of hearing and balance. This early neonatal window of therapeutic efficacy holds irrespective of the molecular strategy, delivery modality, or viral vector serotype deployed. Moreover, a corollary prenatal window of therapeutic efficacy in humans is predicted to close by 19 weeks of GA before the onset of fetal hearing. The efficacy of fetal gene therapy to preemptively correct deleterious genetic mutations in the organogenesis stage mouse embryo before the onset of inner ear disease is an emerging research focus that has promising potential. The process of defining and validating fetal and postnatal gene therapies will be critically enhanced by rigorous evaluation in higher vertebrate model systems in which the fetal onset of hearing is a defining feature.

## Author Contributions

LW, JK and JB identified the need for a comprehensive review of the literature on gene therapy to treat inner ear disease. LW, JK and JB compiled the necessary literature and wrote the manuscript.

## Conflict of Interest Statement

The authors declare that the research was conducted in the absence of any commercial or financial relationships that could be construed as a potential conflict of interest.
